# Reply to: On the difficulty of achieving differential privacy in practice: user-level guarantees in aggregate location data

**DOI:** 10.1038/s41467-021-27567-z

**Published:** 2022-01-10

**Authors:** Aleix Bassolas, Hugo Barbosa-Filho, Brian Dickinson, Xerxes Dotiwalla, Paul Eastham, Riccardo Gallotti, Gourab Ghoshal, Bryant Gipson, Surendra A. Hazarie, Henry Kautz, Onur Kucuktunc, Allison Lieber, Adam Sadilek, Jose J. Ramasco

**Affiliations:** 1 School of Mathematical Sciences, Queen Mary University of London, London, E1 4NS UK; 2 Department of Physics & Astronomy, University of Rochester, Rochester, NY USA; 3 Department of Computer Science, University of Rochester, Rochester, NY USA; 4 Google Inc, 1600 Amphitheatre Parkway, Mountain View, CA USA; 5 Bruno Kessler Foundation (FBK), Trento, Italy; 6 Goergen Institute for Data Science, University of Rochester, Rochester, NY USA; 7 Instituto de Física Interdisciplinar y Sistemas Complejos IFISC (CSIC-UIB), Campus UIB, 07122 Palma de Mallorca, Spain

**Keywords:** Environmental impact, Complex networks

**replying to** F. Houssiau et al. *Nature Communications* 10.1038/s41467-021-27566-0 (2021)

In the work developed in Bassolas et al.^1^, we studied the structure of cities and their impact in city livability using a highly aggregated mobility dataset. In order to protect privacy, random noise was added using an automated Laplace mechanism (*ε*, *δ*)-differential privacy, with *ε* = 0.66 and *δ* = 2.1 × 10^−29^. Where *ε* sets the noise intensity and *δ* stands for the deviation from pure *ε*-privacy.

To illustrate the protection provided by a layer of (*ε*, *δ*)-differential privacy, with *ε* = 0.66 and *δ* = 2.1 × 10^−29^, we note that an attacker can improve their certainty about an individual’s presence or absence in the dataset by at most 16%. This observation holds even if the attacker knows every individual’s data, including that of the target, via some side channel. An attack model like this is known as *membership inference with perfect knowledge*.

In their analysis, Houssiau et al. assume that the dataset referred to in the statistic is the entry dataset of trips. However, we specify the layer of (*ε*, *δ*)-differential privacy as per metric, i.e., *the number of trips from location A to location B per week W*. In other words, the *unit of privacy* that is protected with the promised differential privacy guarantees is not an individual’s contribution to the entire dataset, but rather whether the individual made a trip from A to B during week W. We agree with Houssiau et al. that it is important to communicate privacy protection precisely and we should have been more specific to avoid confusion.

It is worth pointing out that although Houssiau et al. correctly hypothesize that the 16% statistic does not hold when applied to the entire dataset, there are some discrepancies between their analysis and the privacy mechanisms we apply, resulting in stronger privacy protection in practice. In particular, we bound an individual’s contribution to a particular aggregation partition, i.e., trips from A to B within a week W, to 1. Moreover, the geographical areas we consider are grid cells of size ~1.3 km^2^ rather than exact locations, as Houssiau et al. assume. Thus, Houssiau et al.’s analysis of a single user (one of the authors), who reported 39 trips in total, likely translates to fewer contributions to the entire dataset and consequently also results in less privacy loss when evaluated over the entire dataset. Finally, we want to emphasize that membership inference with perfect knowledge of the entire dataset is a very strong attack model that is unrealistic in practice. So we stand by our claim that the dataset is highly aggregated and anonymous for all practical purposes.

Below we provide a clarified description of our data aggregation:

The automated Laplace mechanism adds random noise drawn from a zero mean Laplace distribution and yields (*ε*, *δ*)-differential privacy guarantee of *ε* = 0.66 and *δ* = 2.1 × 10^−29^ per metric. Specifically, for each week W and each location pair (A, B), we compute the number of unique users who took a trip from location A to location B during week W. To each of these metrics, we add Laplace noise from a zero-mean distribution of scale 1/0.66. We then remove all metrics for which the noisy number of users is lower than 100, following the process described in ref. ^2^ and publish those remaining. Each metric published therefore satisfies (*ε*, *δ*)-differential privacy with values defined above.

The parameter *ε* controls the noise intensity in terms of its variance, while *δ* represents the deviation from pure *ε*-privacy. The closer they are to zero, the stronger the privacy guarantees. For example, with these values of the parameters, an attacker with perfect knowledge on all users except user U would increase the level of certainty as to whether U went from geographical area A to area B during a given week no more than 16%. Each user contributes at most one increment to each partition. If they go from a region A to another region B multiple times in the same week, they only contribute once to the aggregation count. No individual user data was ever manually inspected, only heavily aggregated flows of large populations were handled.

## Data Availability

Data sharing is not applicable to this article as no datasets were generated or analyzed during the current study.

## Code availability

Code sharing is not applicable to this article as no datasets were generated or analyzed during the current study.
